# Factors associated with physical inactivity among Palestinians with type 2 diabetes mellitus treated in resource-limited settings

**DOI:** 10.1038/s41598-024-60876-z

**Published:** 2024-05-16

**Authors:** Ramzi Shawahna, Mohammad Jaber, Arob Zmiro, Sewar Kashkoush

**Affiliations:** 1 Department of Physiology, Pharmacology and Toxicology, Faculty of Medicine and Health Sciences, An-Najah National University, New Campus, Building: 19, Office: 1340, Nablus, Palestine; 2 Clinical Research Center, An-Najah National University Hospital, Nablus, 44839 Palestine; 3 Department of Medicine, Faculty of Medicine and Health Sciences, An-Najah National University, Nablus, Palestine; 4 An-Najah National University Hospital, Nablus, 44839 Palestine

**Keywords:** Physical activity, Type 2 diabetes, Health care system, Exercise, Palestine, Diabetes, Public health

## Abstract

This study determined the prevalence and the associated factors with meeting the recommended amount of physical activity among type 2 diabetes mellitus (T2DM) patients receiving care in resource-limited settings of the West Bank of Palestine. Physical activity was assessed using the World Health Organization’s Global Physical Activity Questionnaire. Associations were examined using multivariate logistic regression. Of the 302 patients included, 117 (38.7%) met the recommended amount of physical activity. Being younger than 58 years [aOR = 2.1 (95% CI 1.0–4.3], were employed [aOR = 2.3 (95% CI 1.1–4.9)], had high income [aOR = 3.9 (95% CI 1.3–11.9)], had thought that physical activity was crucial for T2DM patients [aOR = 32.7 (95% CI 3.9–275.5)], did not have comorbidities [aOR = 2.2 (95% CI 1.1–4.4)], had normal weight [aOR = 2.8 (95% CI 1.3–6.0)], and those who were overweight [aOR = 2.6 (95% CI 1.1–6.0)] were more likely to meet the recommended amount of physical activity compared to the patients who were 58 years or older, had low income, did not think that physical activity was crucial for T2DM patients, had comorbidities, and were obese, respectively. There is a need to increase physical activity among T2DM patients in resource limited settings.

## Introduction

Globally, physical inactivity is considered a major cause of significant mortality and morbidity^1,2^. It is well-established that physical inactivity is causally associated with a wide range of negative health outcomes that include cardiovascular diseases, type 2 diabetes mellitus (T2DM), and obesity^3^. According to some estimates, physical inactivity caused 5.3 million deaths globally which accounted for about 9% of all premature mortality in 2008^4^.

The World Health Organization (WHO) has defined physical inactivity as engaging in less than 600 metabolic equivalent tasks (METs) minutes per week^5^. In 2016, the global age-standardized prevalence of physical inactivity was 27.5% (95% uncertainty interval 25.0–32.2) based on 358 studies that were conducted in 168 countries and included a total of 1.9 million participants^6^. The level of physical inactivity can be affected by age, gender, sociodemographic, cultural, health conditions/disabilities, and personal factors^7,8^. It has been estimated that 7.2% of all all-cause mortality among T2DM patients can be attributed to physical inactivity^4^.

Physical inactivity is a modifiable risk factor for metabolic diseases including T2DM. In addition, being physically active was shown to be associated with multiple health benefits^9^. Therefore, many health organizations and professional bodies including the WHO and the World Health Assembly (WHA) have called for higher levels of physical activity and launched initiatives to reduce levels of physical inactivity^10^. The WHO’s revised Global Recommendations on Physical Activity for Health offer fresh guidelines to decrease levels of physical inactivity, emphasizing the message that engaging in any amount of physical activity is preferable to none, and increasing physical activity levels can lead to better health outcomes^10,11^.

Accumulating evidence has shown that T2DM patients can achieve adequate glycemic control using pharmacologic and nonpharmacologic approaches^12,13^. In addition to diet, physical activity is one of the most commonly used nonpharmacological approaches to help achieve adequate glycemic control among T2DM patients. Systematic reviews of different studies showed that T2DM patients, breast cancer, ischemic heart disease, and chronic obstructive pulmonary disease who engaged in higher levels of physical activity after their diagnosis have lower mortality rates^12,14–16^. Additionally, higher levels of physical activity were shown to improve body composition, muscular strength, bone health, cardiorespiratory functions, psychological health, and the quality of life of the patients^12^.

Even though physical activity is a cornerstone in the management of T2DM, several studies have reported suboptimal levels of physical activity among T2DM patients ^12,17–19^. It has been argued that the likelihood of meeting the recommended amount of physical activity among T2DM patients can be influenced by sociodemographic, economic, health-related, and psychological factors. The patients who are old, have other comorbid conditions, and high body mass index often face physical and/or health-related limitations to engage in physical activity^20^. Similarly, employed patients may have limited leisure time that can be used for physical activity, especially when their job is more sedentary^21^. Moreover, patients who live in rural areas and refugee camps and those with low income have limited access to recreational and sports facilities^22,23^. Although married patients could have social support from their spouses to engage in physical activity, they can also enjoy less autonomy in scheduling their physical activities. It has been argued that education can improve health awareness and knowledge of the benefits of physical activity for T2DM patients^24^. A few studies were conducted to assess the prevalence of physical inactivity among Palestinians with T2DM. These studies were conducted in the Gaza Strip^25–27^. Physical inactivity levels were high among T2DM patients in the Gaza Strip. However, little is known about the prevalence of physical inactivity among T2DM patients in the West Bank, notably those receiving care in resource-limited settings^28^. We hypothesized that similar to the data reported from the Gaza Strip, the prevalence of physical inactivity among T2DM patients in the West Bank was also high. There was a need to determine the associations between the sociodemographic and clinical factors of the patients and meeting the recommended amount of physical activity. Therefore, this study was conducted to assess the prevalence of T2DM patients meeting the recommended amount of physical activity and to determine the associations between different sociodemographic and clinical factors and meeting the recommended amount of physical activity among T2DM patients who received care in resource-limited settings in the West Bank of Palestine.

## Methods

### Study area

**Figure 1:**
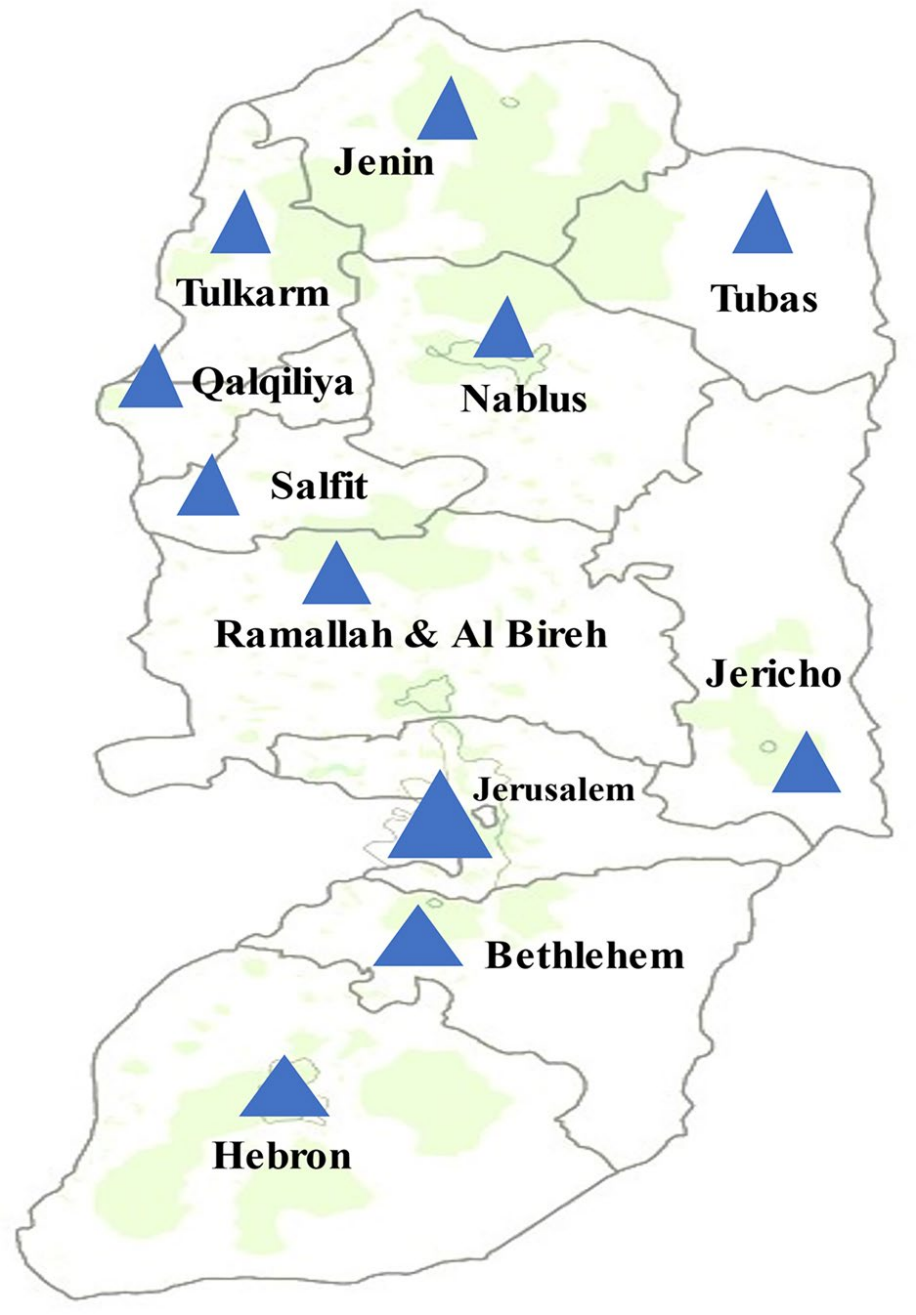
Map of the West Bank showing the governorates (the map was modified from Wikimedia Commons: www.commons.wikimedia.org/wiki).

This study was conducted in the resource-limited primary healthcare clinics distributed across the different governorates in the West Bank including those in the refugee camps. Figure 1 shows a map of the West Bank with the different governorates.

### Study design and participants

The study was carried out using a cross-sectional approach. The study adhered to the guidelines of the STROBE (Strengthening the Reporting of Observational Studies in Epidemiology) statement^29^.

The participants in this study were T2DM patients who received care in the Palestinian healthcare system. The necessary sample size was determined based on the number of individuals in Palestine with T2DM, as reported by the Palestinian Ministry of Health. At the time of the research, the number of individuals with diabetes mellitus in Palestine was 270,465, with 95.3% of those having T2DM. An online calculator (Raosoft, Raosoft, Inc.) was used to calculate the appropriate sample size with a 95% confidence level and a margin of error of 5% using physical activity data (time spent in moderate to vigorous physical activity) reported in previous studies among T2DM patients^8,30–37^. The calculated sample size was 384 patients. In this study, we decided to invite 400 T2DM patients.

The patients were included in this study using a convenience sampling approach when they met the following inclusion criteria: (1) being 18 years and older, (2) having been diagnosed with T2DM, (3) received healthcare services in the Palestinian healthcare system, (4) willing to respond to items in a questionnaire, and (5) providing written informed consent. Patients who had a record of mental and cognitive impairments or as diagnosed by physicians were excluded from this study.

### Physical activity outcome

The total physical activity time and METs were the dependent outcomes measured in this study. It is noteworthy mentioning that physical activity time and METs are related concepts. However, the two concepts differ in measuring physical exertion and energy expenditure^33–35^. While physical activity refers to any bodily movement that requires expenditure of energy, METs are a measure of the energy expenditure while performing the different forms of physical activity. One MET is defined as the energy expenditure while resting. This is equivalent to the amount of energy expenditure while sitting quietly. The different forms of physical activity performed are assigned MET values based on their intensity relative to the metabolic rate at resting. For instance, running or performing a vigorous exercise has a higher MET value compared to walking at a moderate pace.

The total physical activity time and MET-minutes/week were measured using the WHO’s Global Physical Activity Questionnaire (GPAQ)^33^. Using 16 items, the GPAQ measured the physical activity time and MET-minutes/week performed at work, traveling to and from places, and during recreational activities. The GPAQ was shown to be valid and reliable in measuring the amount of physical activity time and MET-minutes/week in different populations and countries^34,35^. As guided by the GPAQ analysis manual, the patients were classified into MET classes as: (1) low: < 3 METs (< 600 MET-minutes/week), (2) moderate: 3–6 METs (600–3000 MET-minutes/week), and (3) high: > 6 METs (> 3000 MET-minutes/week)^34^. According to the WHO, one should expend at least 600 MET-minutes/week to meet the recommended amount of physical activity^5^.

Compared to other tools that can be used to measure physical activity and energy expenditure, the WHO’s GPAQ was preferred because it was more often used to assess physical activity levels in low- and middle-income countries^38–40^.

### Independent variables

For this research, a questionnaire specifically designed for the study was utilized. Previous studies that assessed physical activity levels in individuals with diabetes were consulted while developing of the questionnaire^8,36,37^. While developing the questionnaire, the literature was searched for the factors that could affect meeting the recommended amount of physical activity. Meeting the recommended amount of physical activity was influenced by sociodemographic and illness-related factors^34,35,38–40^. To determine the associations between the different sociodemographic and clinical factors and meeting the recommended amount of physical activity among T2DM patients in this study, variables like age, marital status, employment status, place of residence, educational attainment, monthly household income, medical insurance status, and type of medical insurance were collected. The patients were also asked to indicate whether they have participated in an awareness program related to T2DM, received education about the importance of physical activity in T2DM management, were physically active before being diagnosed with T2DM, and whether they thought that physical activity was beneficial for T2DM patients. In addition, the patients were asked to report if they had hyperlipidemia, hypertension, or other chronic health conditions, and the time elapsed since they received their diagnosis with T2DM. The body mass index of the patients was calculated based on their weight and height. The last glycosylated hemoglobin (HbA_1c_), fasting plasma glucose, and postprandial plasma glucose levels were obtained from their medical records.

### Statistical analysis

The data were analyzed using IBM SPSS v. 21.0 (IBM Inc., Armonk, NY). The collected data were tested for normal distribution using the Shapiro–Wilk test, which revealed that the data did not conform to a normal distribution. Therefore, medians with the corresponding interquartile range [IQR = first (Q1) and third (Q3) quartiles] were employed. Differences in the physical activity time and MET-minutes/week as continuous variables were assessed using the Mann–Whitney U and Kruskal–Wallis tests. The correlations between the continuous variables were assessed using Spearman's rank correlations. Low, moderate, and high METs classes were described using frequencies (n) and percentages (n). The associations between demographic and clinical variables of the patients with the three METs classes (low, moderate, and high) were investigated using Chi-square tests. Because meeting the recommended amount of physical activity was indicated by expending 600 or more MET-minutes/week^5^, the patients were categorized into two groups: those who did not meet the recommended amount of physical activity (< 3 METs (< 600 MET-minutes/week)) and those who met the recommended amount of physical activity ((≥ 3METs (≥ 600 MET-minutes/week). The continuous variables like age, HbA_1c_, fasting plasma glucose level, and postprandial plasma glucose level were categorized around their medians. The body mass index was categorized as: normal weight < 25 kg/m^2^, overweight: 25–29.9 kg/m^2^, or obese: ≥ 30 kg/m^2^. The household income was categorized as: low (< US$ 800), medium (US$ 800–1350), or high (> US$ 1350) based on the reports of the Palestinian Central Bureau of Statistics. The associations between demographic and clinical variables of the patients with meeting or not meeting the recommended amount of physical activity were investigated using Chi-square tests. A multivariate logistic regression model was used to calculate the adjusted odds ratios (aOR) for meeting the recommended amount of physical activity. All independent variables were retained in the multivariate logistic regression model. A* p*-value of < 0.05 indicated statistical significance.

### Ethical approvals

This study adhered to both local and international ethical principles governing scientific research involving human subjects. The research received approval from the Institutional Review Board (IRB) of An-Najah National University and also obtained consent from the Health Education Office of the Ministry of Health. Written informed consent was provided by all patients, and hospital and clinic managers approved the researchers' access to the patient's medical records. The data collected were coded and analyzed anonymously to protect the patients' privacy.

## Results

Of the 400 invited patients, 302 completed the questionnaire, giving a response rate of 75.5%. The median age of the patients was 58.0 [IQR = 50.0, 67.0] years and a median time since diagnosis of 9.0 [IQR = 5.0, 15.0] years. The median body mass index was 28.1 [IQR = 24.9, 33.1] kg/m^2^, while the median fasting plasma glucose level was 170.0 [IQR = 130.0, 230.0] mg/dL, the median postprandial plasma glucose level was 220.0 [IQR = 170.0, 300.0] mg/dL, and the median HbA_1c_ was 8.0% [IQR = 7.0%, 10.0%]. The detailed information on the patient's sociodemographic and clinical characteristics are shown in Table 1.

**Table 1:** Detailed sociodemographic and disease characteristics of the patients.

Variable	n	%
**Age (years)**		
< 58	139	46.0
≥ 58	163	54.0
Marital status		
Single	29	9.6
Married	246	81.5
Divorced/widowed	27	8.9
Employment status		
Unemployed	160	53.0
Employed	142	47.0
Place of residence		
City	161	53.3
Village	111	36.8
Refugee camp	30	9.9
Educational level		
School	216	71.5
Undergraduate	73	24.2
Postgraduate	13	4.3
Household monthly income		
Low (< US$ 800)	191	63.2
Medium (US$ 800- US$ 1,350)	78	25.8
High (> US$ 1,350)	33	10.9
Medical insurance status		
No	3	1.0
Yes	299	99.0
Type of medical insurance*		
Governmental	277	91.7
Private	22	7.3
Participated in an awareness program related to T2DM		
No	167	55.3
Yes	132	43.7
Received education about the importance of physical activity in T2DM management		
No	145	48.0
Yes	154	51.0
Physically active before diagnosis		
No	161	53.3
Yes	138	45.7
Thinking that physical activity is crucial in T2DM patients		
No	47	15.6
Yes	252	83.4
Have hyperlipidemia		
No	117	38.7
Yes	182	60.3
Have hypertension		
No	107	35.4
Yes	192	63.6
Have other health conditions		
No	187	61.9
Yes	112	37.1
Time since diagnosis (years)		
< 10	157	52.0
≥ 10	142	47.0
Body mass index class		
Normal weight (< 25 kg/m^2^)	79	26.2
Overweight (25–29.9 kg/m^2^)	101	33.4
Obese (≥ 30 kg/m^2^)	122	40.4
HbA_1c_ (%)		
< 7	58	19.2
≥ 7	241	79.8
Fasting plasma glucose level (mg/dL)		
< 130	61	20.2
≥ 130	241	79.8
Postprandial plasma glucose level (mg/dL)		
< 180	81	26.8
≥ 180	221	73.2

*Calculated based on the number of patients who had health insurance, T2DM: type 2 diabetes mellitus, HbA_1c_: hemoglobin A_1c_.

### Responses of the patients on the global physical activity questionnaire items

In this study, many patients reported low levels of physical activity at work, during travel to and from places, and during recreational activities. The detailed responses of the patients on the items in the Global Physical Activity Questionnaire are shown in Supplementary Table S1.

### Associations between physical activity outcome and the independent variables

The median physical activity was 480.0 [IQR = 69.0, 1440.0] min and the median MET-min/week was 100.0 [IQR = 0.0, 272.5]. The total physical activity and MET were significantly higher for patients who were younger than 58 years, employed, had a university degree, had median or high income, had participated in an awareness program related to T2DM, had received education about the importance of physical activity in T2DM management, were physically active before being diagnosed with T2DM, thought that physical activity was crucial for T2DM patients, did not have hyperlipidemia, hypertension, or other health conditions, was diagnosed with T2DM less than 10 years ago, had normal body weight, had HbA_1c_ less than 7%, had a fasting plasma glucose level less than 130 mg/dL, and had a postprandial plasma glucose level less than 180 mg/dL. Details of the differences in total physical activity and MET in relation to the demographic and disease variables of the patients are shown in Supplementary Table S2.

When the patients were stratified into MET classes, 185 (61.3%) of the patients reported low physical activity and 11.9% reported high physical activity. Significantly more patients were in the high MET class when they were younger than 58 years, employed, had a university education, had medium or higher income, had participated in an awareness program related to T2DM, had received education about the importance of physical activity in T2DM management, were physically active before being diagnosed with T2DM, thought that physical activity was crucial for T2DM patients, did not have hyperlipidemia, hypertension, or other health conditions, was diagnosed with T2DM less than 10 years ago, had normal body weight, had HbA_1c_ less than 7%, had a fasting plasma glucose level less than 130 mg/dL, and had a postprandial plasma glucose level less than 180 mg/dL. Details of the associations are shown in Supplementary Table S3.

When the patients were stratified into meeting or not meeting the recommended amount of physical activity, 117 (38.7%) patients met the recommended amount of physical activity. Chi-square tests showed that meeting the recommended amount of physical activity was significantly associated with younger age, being employed, having a university degree, and having higher income (Table 2). In addition, having participated in an awareness program, having received education about the importance of physical activity in T2DM management, being physically active before diagnosis with T2DM, and thinking that physical activity was crucial for T2DM patients were also associated with meeting the recommended amount of physical activity. Moreover, patients who did not have hyperlipidemia, hypertension, or other health conditions, were diagnosed with T2DM less than 10 years ago, had normal body weight, had HbA_1c_ less than 7%, had a fasting plasma glucose level less than 130 mg/dL, and had a postprandial plasma glucose level less than 180 mg/dL were more likely to meet the recommended amount of physical activity. Details of the associations are shown in Table 2.

**Table 2:** Associations between demographic and disease variables of the patients with meeting or not meeting the recommended amount of physical activity.

Variable	Meeting the recommended amount of physical activity*								
	No		Yes		*p*-value	aOR	95% CI for OR		*p*-value
	n	%	n	%			Lower	Upper	
Age (years)									
< 58	60	19.9	79	26.2	< 0.001	2.1	1.0	4.3	0.050
≥ 58	125	41.4	38	12.6		Reference			
Marital status									
Single	17	5.6	12	4.0	0.583	1.8	0.4	7.8	0.424
Married	149	49.3	97	32.1		1.8	0.6	5.7	0.287
Divorced/widowed	19	6.3	8	2.6		Reference			
Employment status									
Unemployed	127	42.1	33	10.9	< 0.001	Reference			
Employed	58	19.2	84	27.8		2.3	1.1	4.9	0.032
Place of residence									
City	95	31.5	66	21.9	0.335	1.4	0.4	4.4	0.594
Village	68	22.5	43	14.2		1.1	0.3	3.5	0.882
Refugee camp	22	7.3	8	2.6		Reference			
Educational level									
School	151	50.0	65	21.5	< 0.001	Reference			
Undergraduate	28	9.3	45	14.9		0.7	0.1	3.1	0.594
Postgraduate	6	2.0	7	2.3		1.1	0.2	4.9	0.913
Household monthly income									
Low (< US$ 800)	141	46.7	50	16.6	< 0.001	Reference			
Medium (US$ 800- US$ 1,350)	27	8.9	51	16.9		3.2	0.9	11.3	0.063
High (> US$ 1350)	17	5.6	16	5.3		3.9	1.3	11.9	0.018
Type of medical insurance**									
Governmental	174	57.6	103	34.1	0.107	Reference			
Private	10	3.3	12	4.0		1.4	0.4	4.4	0.563
Participated in an awareness program related to T2DM									
No	125	41.4	44	14.6	< 0.001	Reference			
Yes	60	19.9	73	24.2		1.0	0.5	2.2	0.910
Received education about the importance of physical activity in T2DM management									
No	108	35.8	38	12.6	< 0.001	Reference			
Yes	77	25.5	79	26.2		1.1	0.5	2.2	0.826
Physically active before diagnosis									
No	117	38.7	47	15.6	< 0.001	Reference			
Yes	68	22.5	70	23.2		1.7	0.9	3.2	0.123
Thought that physical activity was crucial for patients with T2DM									
No	47	15.6	1	0.3	< 0.001	Reference			
Yes	138	45.7	116	38.4		32.7	3.9	275.5	0.001
Have hyperlipidemia									
No	51	16.9	68	22.5	< 0.001	1.2	0.6	2.7	0.593
Yes	134	44.4	49	16.2		Reference			
Have hypertension									
No	41	13.6	68	22.5	< 0.001	1.3	0.6	2.8	0.562
Yes	144	47.7	49	16.2		Reference			
Have other health conditions									
No	94	31.1	96	31.8	< 0.001	2.2	1.1	4.4	0.025
Yes	91	30.1	21	7.0		Reference			
Time since diagnosis (years)									
< 10	78	25.8	80	26.5	< 0.001	1.0	0.5	2.1	0.981
≥ 10	107	35.4	37	12.3		Reference			
Body mass index class									
Normal weight (< 25 kg/m^2^)	34	11.3	45	14.9	< 0.001	2.8	1.3	6.0	0.010
Overweight (25–29.9 kg/m^2^)	53	17.5	48	15.9		2.6	1.1	6.0	0.031
Obese (≥ 30 kg/m^2^)	98	32.5	24	7.9		Reference			
HbA_1c_ (%)									
< 7	25	8.3	33	10.9	0.002	1.7	0.7	4.1	0.236
≥ 7	160	53.0	84	27.8		Reference			
Fasting plasma glucose level (mg/dL)									
< 130	24	7.9	37	12.3	< 0.001	1.1	0.4	3.3	0.830
≥ 130	161	53.3	80	26.5		Reference			
Postprandial plasma glucose level (mg/dL)									
< 180	32	10.6	49	16.2	< 0.001	1.0	0.4	2.8	0.954
≥ 180	153	50.7	68	22.5		Reference			

*The patients with (< 3 METs (< 600 MET-minutes/week)) were considered not to have met the recommended amount of physical activity and those with ((≥ 3METs (≥ 600 MET-minutes/week) were considered to have met the recommended amount of physical activity, **Calculated based on the number of patients who had health insurance, aOR: adjusted odds ratio, T2DM: type 2 diabetes mellitus, HbA_1c_: hemoglobin A_1c_.

The multivariate logistic regression model showed that the odds of the patients who were younger than 58 years were 2.1-fold (95% CI 1.0–4.3) more likely to meet the recommended amount of physical activity compared to the patients who were 58 years or older (Table 2). The odds for the patients who were employed were 2.3-fold (95% CI 1.1–4.9) more likely to meet the recommended amount of physical activity compared to the patients who unemployed. The odds for the patients who had high household income were 3.9-fold (95% CI 1.3–11.9) more likely to meet the recommended amount of physical activity compared to the patients who had low household income. The odds for the patients who had thought that physical activity was crucial for T2DM patients were 32.7-fold (95% CI 3.9–275.5) more likely to meet the recommended amount of physical activity compared to the patients who did not think that physical activity was crucial for T2DM patients. The odds for the patients who did not have comorbidities were 2.2-fold (95% CI 1.1–4.4) more likely to meet the recommended amount of physical activity compared to the patients who had comorbidities. The odds for the patients who had normal weight and those who were overweight were 2.8-fold (95% CI 1.3–6.0) and 2.6-fold (95% CI 1.1–6.0) more likely to meet the recommended amount of physical activity compared to the patients who were obese, respectively. Details of the multivariate logistic regression model are shown in Table 2.

There was a significant negative correlation between total physical activity and MET with age, the time elapsed since diagnosis, body mass index, fasting plasma glucose level, postprandial plasma glucose level, and HbA_1c_. The correlation matrix is shown in Supplementary Table S4.

## Discussion

Evidence of the benefits of physical activity for T2DM patients is well-established. Adherence to the recommended amount of physical activity was shown to improve insulin sensitivity, help achieve glycemic control, improve cardiorespiratory fitness, blood pressure control, weight loss, lipid profile, psychological health, and quality of life of the patients^12^. For the first time, levels of physical activity were assessed among T2DM patients in the West Bank. Additionally, the factors associated with meeting the recommended amount of physical activity were also investigated. The findings reported in this study would be informative to decision and policymakers who might be interested in designing appropriate measures to increase physical activity and reduce physical inactivity among T2DM patients.

In this study, the majority (61.3%) of the T2DM patients reported low physical activity. Moreover, only 38.7% of the patients met the recommended amount of physical activity. The findings reported in this study were consistent with those reported in the Arab region^8,25,26,41,42^. Additionally, the findings were also consistent with those reported in the Western world including the US in which more than 60% of T2DM patients were reportedly inactive^43^. Given the increasing consumption of energy-rich diets and adopting sedentary lifestyles, countries in the Middle East and North Africa (MENA) are expected to have a 96% increase in the number of patients with diabetes by the year 2035^44^. Taken together these findings indicate that there is a pressing need to design effective measures to increase physical activity and reduce sedentary lifestyles among T2DM patients.

Although 27.2% of the patients reported no physical activity in this study, this percentage was lower than those reported in previous studies like in Oman (60.3%) and the US^8,45^. Similar to previous studies, the patients who reported low physical activity had higher HbA_1c_, fasting plasma glucose, and postprandial plasma glucose levels. These findings were not surprising as the benefits of physical activity in helping patients achieve glycemic control were well-established^12^.

The findings of this study showed that higher physical activity was associated with younger age, employment, high income, positive attitudes toward physical activity, reporting the absence of comorbidities, and not being obese. Similarly, higher recreational inactivity was also reported among patients who lacked financial resources^34^. The results reported in this study were consistent with those reported in other countries including Arab countries^8,25,26,34,41,42,46–49^. Old age and high body mass index were predictors of physical inactivity. It was estimated that travel inactivity increased by 6% for every increased unit of body mass index. Younger patients without existing chronic comorbidities would likely face fewer barriers to performing physical activities compared to older patients with chronic diseases^8^. Probably, those patients would find it easier to travel and practice recreational activities compared to older patients who have other chronic comorbidities^50^. Healthcare providers should consider providing more comprehensive healthcare services to T2DM patients in the primary healthcare clinics. In addition, T2DM patients should be assessed and monitored for complications. Moreover, comorbidities, HbA_1c_, body mass index, blood pressure, and lipid profile should be periodically assessed and appropriate recommendations should be made. Probably, healthcare providers should consider counseling older patients on the importance of physical activity and maintaining a healthy weight. Suitable physical activity programs should be developed and tailored to the needs of the individual patients. Physical activity programs might consider avoiding the use of expensive exercise equipment. These programs might include walking, gardening, and home-based exercise programs that require minimal or no equipment. Moreover, the patients who expressed positive attitudes toward physical activity were more likely to report higher physical activity. These findings might indicate that education, awareness, and attitude changing interventions might be effective in increasing physical activity and decreasing physical inactivity. These findings indicate that decision- and policymakers should consider intensifying campaigns and initiatives that target patients who are older, obese, have comorbidities, and have negative attitudes toward the benefits of physical activity for T2DM patients.

The findings reported in this study are consideration worthy as the Palestinian healthcare system is complex and highly fragmented. T2DM patients receive healthcare services in healthcare clinics that belong to the governmental sector, the non-governmental organizations, the Palestinian Military Medical Services, the United Nations Relief and Work Agency, and the private sector^51^. It is noteworthy to mention that none of these sectors provide comprehensive healthcare services. The Palestinian Ministry of Health provides primary healthcare services to T2DM patients through primary healthcare clinics that are distributed throughout the West Bank of Palestine^51,52^. The healthcare professionals providing services through these clinics reported lack of training on the care guidelines^52^. In addition, the clinics lacked essential equipment to assess for diabetic complications including ophthalmoscopes, visual acuity charts, electrocardiogram machines, tuning forks, and foot-care equipment. Moreover, weight and height measurements, calculation of body mass index, and measurement of blood pressure were often neglected. Many clinics do not offer monofilament, microalbumin, and glycated hemoglobin A_1c_ (HbA_1c_) tests. Similarly, nutrition and physical activity specialists are not available in many of these clinics. The consultations with physicians and nurses are often short, not individualized, or recorded. The appointment system and protocols for periodic monitoring are often not used. Decision-makers in the healthcare authorities might use the findings reported in this study to improve the care services provided to T2DM patients in these clinics.

### Strengths and limitations

The findings of this study should be interpreted in light of the following strengths and limitations. First, this was the first study in the West Bank to determine the level of physical activity among T2DM patients. The factors associated with low, moderate, and high physical activity were also determined. The findings of this study could be informative to decision and policymakers in the Palestinian healthcare authorities and similar healthcare systems. Second, the study was conducted using a robust tool that was previously validated and tested for reliability in different settings and countries. The use of this robust, valid, and reliable tool should have promoted comparability of the findings with those reported in other studies. Third, the sample of patients who were included in this study was diversified in terms of demographic and disease variables. This diversity should have improved the representativeness of the entire population of T2DM patients and should have improved the external validity of the findings reported in this study.

On the other hand, the findings of this study should also be interpreted after considering the following limitations. First, the study was conducted in a cross-sectional design. Cross-sectional studies are not suitable to study causalities. Second, many of the data collected in this study were self-reported. Therefore, social desirability and recall bias cannot be excluded. Third, the sample size used in this study was not very large compared to previous studies. A larger sample size could have allowed a more solid conclusion.

## Conclusion

T2DM patients who were included in this study reported low physical activity as determined by the WHO’s GPAQ. Higher physical activity was associated with younger age, high income, positive attitudes toward physical activity, absence of comorbidities, and not being obese. Decision and policymakers in healthcare authorities need to consider designing effective interventions to increase physical activity and reduce physical inactivity among T2DM patients in resource limited settings. More studies are still needed to determine which interventions are superior in increasing physical activity among T2DM patients.

### Supplementary Information

Supplementary Tables.

## Data Availability

All data relevant to this study were included in the “Results” section of this manuscript.
