# Supplementary material for: Factors associated with physical inactivity among Palestinians with type 2 diabetes mellitus treated in resource-limited settings
Source: Sci Rep. 2024 May 16;14:11256. doi: 10.1038/s41598-024-60876-z (PMC11099123; doi:10.1038/s41598-024-60876-z)
Supplement: Supplementary file 1 — Supplementary Tables. [file 41598_2024_60876_MOESM1_ESM.docx]

**Supplementary materials for the manuscript**

**Factors associated with physical inactivity among Palestinians with type 2 diabetes mellitus treated in resource-limited settings**

Ramzi Shawahna^1,2*^, Mohammad Jaber^3,4^, Arob Zmiro^3^, Sewar Kashkoush^3^

^1^Department of Physiology, Pharmacology and Toxicology, Faculty of Medicine and Health Sciences, An-Najah National University, Nablus, Palestine

^2^Clinical Research Center, An-Najah National University Hospital, Nablus, 44839, Palestine

^3^Department of Medicine, Faculty of Medicine and Health Sciences, An-Najah National University, Nablus, Palestine

^4^An-Najah National University Hospital, Nablus, 44839, Palestine

**^*^Correspondence:**

Ramzi Shawahna, PhD, Department of Physiology, Pharmacology and Toxicology, Faculty of Medicine & Health Sciences, New Campus, Building: 19, Office: 1340, An-Najah National University, P.O. Box 7, Nablus, Palestine

Phone: + (970) 923 45113 ext 2772

Phone: + (970) 92349739

Email: [ramzi_shawahna@hotmail.com](mailto:ramzi_shawahna@hotmail.com)

**Supplementary Table S1:** Detailed responses of the patients on the items in the Global Physical Activity Questionnaire

|  |  | **No** | | **Yes** | |  |  |  |
| --- | --- | --- | --- | --- | --- | --- | --- | --- |
| **#** | **Activity at work** | **n** | **%** | **n** | **%** | **Q1** | **Median** | **Q3** |
| 1 | Does your work involve vigorous-intensity activity that causes large increases in breathing or heart rate like [carrying or lifting heavy loads, digging or construction work] for at least 10 minutes continuously? | 266 | 88.1 | 36 | 11.9 |  |  |  |
| 2 | In a typical week, on how many days do you do vigorous-intensity activities as part of your work? (days) |  |  |  |  | 2.0 | 4.0 | 5.0 |
| 3 | How much time do you spend doing vigorous-intensity activities at work on a typical day? (minutes) |  |  |  |  | 15.0 | 30.0 | 48.0 |
| 4 | Does your work involve moderate-intensity activity that causes small increases in breathing or heart rate such as brisk walking [or carrying light loads] for at least 10 minutes continuously? | 243 | 80.5 | 59 | 19.5 |  |  |  |
| 5 | In a typical week, on how many days do you do moderate-intensity activities as part of your work? |  |  |  |  | 2.0 | 4.0 | 5.0 |
| 6 | How much time do you spend doing moderate-intensity activities at work on a typical day? (minutes) |  |  |  |  | 20.0 | 35.0 | 90.0 |
|  | **Travel to and from places** |  |  |  |  |  |  |  |
| 7 | Do you walk or use a bicycle (pedal cycle) for at least 10 minutes continuously to get to and from places? | 91 | 30.1 | 211 | 69.9 |  |  |  |
| 8 | In a typical week, on how many days do you walk or bicycle for at least 10 minutes continuously to get to and from places? (days) |  |  |  |  | 3.0 | 4.0 | 7.0 |
| 9 | How much time do you spend walking or bicycling for travel on a typical day? (minutes) |  |  |  |  | 20.0 | 35.0 | 60.0 |
|  | **Recreational activities** |  |  |  |  |  |  |  |
| 10 | Do you do any vigorous-intensity sports, fitness or recreational (leisure) activities that cause large increases in breathing or heart rate like [running or football] for at least 10 minutes continuously? | 273 | 90.4 | 29 | 9.6 |  |  |  |
| 11 | In a typical week, on how many days do you do vigorous-intensity sports, fitness or recreational (leisure) activities? |  |  |  |  | 1.0 | 2.0 | 2.0 |
| 12 | How much time do you spend doing vigorous-intensity sports, fitness or recreational activities on a typical day? (hours) |  |  |  |  | 1.0 | 1.5 | 2.0 |
| 13 | Do you do any moderate-intensity sports, fitness or recreational (leisure) activities that causes a small increase in breathing or heart rate such as brisk walking, (cycling, swimming, volleyball) for at least 10 minutes continuously? | 261 | 86.4 | 41 | 13.6 |  |  |  |
| 14 | In a typical week, on how many days do you do moderate-intensity sports, fitness or recreational (leisure) activities? (days) |  |  |  |  | 2.0 | 3.0 | 7.0 |
| 15 | How much time do you spend doing moderate-intensity sports, fitness or recreational (leisure) activities on a typical day? (minutes) |  |  |  |  | 20.0 | 30.0 | 55.0 |
| 16 | How much time do you usually spend sitting or reclining on a typical day? (hours) |  |  |  |  | 3.0 | 7.0 | 10.0 |

**Supplementary Table S2:** Associations between demographic and disease variables of the patients with the total physical activity and MET

|  |  |  | **Total physical activity (min)** | | | | **MET (minutes/week)** | | | |
| --- | --- | --- | --- | --- | --- | --- | --- | --- | --- | --- |
| **Variable** | **n** | **%** | **Q1** | **Median** | **Q3** | **p-value** | **Q1** | **Median** | **Q3** | **p-value** |
| **Age (years)** |  |  |  |  |  |  |  |  |  |  |
| < 58 | 139 | 46.0 | 360.0 | 880.0 | 1920.0 | < 0.001 | 70.0 | 195.0 | 400.0 | < 0.001 |
| ≥ 58 | 163 | 54.0 | 0.0 | 240.0 | 720.0 |  | 0.0 | 60.0 | 140.0 |  |
| **Marital status** |  |  |  |  |  |  |  |  |  |  |
| Single | 29 | 9.6 | 0.0 | 600.0 | 1260.0 | 0.679 | 0.0 | 120.0 | 260.0 | 0.681 |
| Married | 246 | 81.5 | 80.0 | 480.0 | 1464.0 |  | 0.0 | 100.0 | 300.0 |  |
| Divorced/widowed | 27 | 8.9 | 0.0 | 280.0 | 808.0 |  | 0.0 | 60.0 | 195.0 |  |
| **Employment status** |  |  |  |  |  |  |  |  |  |  |
| Unemployed | 160 | 53.0 | 0.0 | 240.0 | 675.0 | < 0.001 | 0.0 | 60.0 | 147.5 | < 0.001 |
| Employed | 142 | 47.0 | 400.0 | 848.0 | 1920.0 |  | 67.5 | 180.0 | 367.5 |  |
| **Place of residence** |  |  |  |  |  |  |  |  |  |  |
| City | 161 | 53.3 | 8.0 | 480.0 | 1380.0 | 0.513 | 0.0 | 120.0 | 300.0 | 0.603 |
| Village | 111 | 36.8 | 120.0 | 420.0 | 1480.0 |  | 0.0 | 100.0 | 270.0 |  |
| Refugees camp | 30 | 9.9 | 45.0 | 264.0 | 1050.0 |  | 0.0 | 60.0 | 198.8 |  |
| **Educational level** |  |  |  |  |  |  |  |  |  |  |
| School | 216 | 71.5 | 0.0 | 300.0 | 984.0 | < 0.001 | 0.0 | 70.0 | 198.8 | < 0.001 |
| Undergraduate | 73 | 24.2 | 480.0 | 856.0 | 1680.0 |  | 120.0 | 200.0 | 365.0 |  |
| Postgraduate | 13 | 4.3 | 60.0 | 1260.0 | 2590.0 |  | 15.0 | 315.0 | 637.5 |  |
| **Household monthly income** |  |  |  |  |  |  |  |  |  |  |
| Low (< 800 US$) | 191 | 63.2 | 0.0 | 280.0 | 840.0 | < 0.001 | 0.0 | 70.0 | 180.0 | < 0.001 |
| Medium (800-1,350 US$) | 78 | 25.8 | 480.0 | 1140.0 | 1800.0 |  | 80.0 | 210.0 | 360.0 |  |
| High (> 1,350 US$) | 33 | 10.9 | 156.0 | 720.0 | 2296.0 |  | 0.0 | 150.0 | 465.0 |  |
| **Type of medical insurance*** |  |  |  |  |  |  |  |  |  |  |
| Governmental | 277 | 91.7 | 40.0 | 420.0 | 1440.0 | 0.356 | 0.0 | 90.0 | 270.0 | 0.205 |
| Private | 22 | 7.3 | 108.0 | 750.0 | 1182.0 |  | 22.5 | 187.5 | 292.5 |  |
| **Participated in an awareness program related to T2DM** |  |  |  |  |  |  |  |  |  |  |
| No | 167 | 55.3 | 0.0 | 280.0 | 720.0 | < 0.001 | 0.0 | 60.0 | 180.0 | < 0.001 |
| Yes | 132 | 43.7 | 360.0 | 840.0 | 1680.0 |  | 60.0 | 180.0 | 360.0 |  |
| **Received education about the importance of physical activity in T2DM management** |  |  |  |  |  |  |  |  |  |  |
| No | 145 | 48.0 | 0.0 | 280.0 | 790.0 | < 0.001 | 0.0 | 60.0 | 187.5 | < 0.001 |
| Yes | 154 | 51.0 | 270.0 | 726.0 | 1607.0 |  | 54.5 | 150.0 | 302.5 |  |
| **Physically active before diagnosis** |  |  |  |  |  |  |  |  |  |  |
| No | 161 | 53.3 | 0.0 | 280.0 | 820.0 | < 0.001 | 0.0 | 60.0 | 180.0 | < 0.001 |
| Yes | 138 | 45.7 | 280.0 | 756.0 | 1680.0 |  | 60.0 | 167.5 | 367.5 |  |
| **Thinking that physical activity was crucial for patients with T2DM** |  |  |  |  |  |  |  |  |  |  |
| No | 47 | 15.6 | 0.0 | 0.0 | 100.0 | < 0.001 | 0.0 | 0.0 | 25.0 | < 0.001 |
| Yes | 252 | 83.4 | 240.0 | 606.0 | 1550.0 |  | 40.0 | 120.0 | 300.0 |  |
| **Have hyperlipidemia** |  |  |  |  |  |  |  |  |  |  |
| No | 117 | 38.7 | 480.0 | 960.0 | 1920.0 | < 0.001 | 80.0 | 180.0 | 380.0 | < 0.001 |
| Yes | 182 | 60.3 | 0.0 | 240.0 | 785.0 |  | 0.0 | 60.0 | 180.0 |  |
| **Have hypertension** |  |  |  |  |  |  |  |  |  |  |
| No | 107 | 35.4 | 480.0 | 960.0 | 1920.0 | < 0.001 | 90.0 | 195.0 | 405.0 | < 0.001 |
| Yes | 192 | 63.6 | 0.0 | 260.0 | 800.0 |  | 0.0 | 60.0 | 180.0 |  |
| **Have other health conditions** |  |  |  |  |  |  |  |  |  |  |
| No | 187 | 61.9 | 240.0 | 720.0 | 1680.0 | < 0.001 | 30.0 | 150.0 | 360.0 | < 0.001 |
| Yes | 112 | 37.1 | 0.0 | 240.0 | 480.0 |  | 0.0 | 60.0 | 116.3 |  |
| **Time since diagnosis (years)** |  |  |  |  |  |  |  |  |  |  |
| < 10 | 157 | 52.0 | 294.0 | 720.0 | 1604.0 | < 0.001 | 35.0 | 150.0 | 300.0 | < 0.001 |
| ≥ 10 | 142 | 47.0 | 0.0 | 240.0 | 840.0 |  | 0.0 | 60.0 | 187.5 |  |
| **Body mass index class (kg/m^2^)** |  |  |  |  |  |  |  |  |  |  |
| Normal weight | 79 | 26.2 | 464.0 | 880.0 | 1960.0 | < 0.001 | 75.0 | 175.0 | 360.0 | < 0.001 |
| Overweight | 101 | 33.4 | 90.0 | 640.0 | 1680.0 |  | 0.0 | 120.0 | 392.5 |  |
| Obese | 122 | 40.4 | 0.0 | 240.0 | 500.0 |  | 0.0 | 60.0 | 125.0 |  |
| **HbA_1c_ (%)** |  |  |  |  |  |  |  |  |  |  |
| < 7 | 58 | 19.2 | 390.0 | 1020.0 | 2010.0 | < 0.001 | 56.3 | 180.0 | 392.5 | 0.006 |
| ≥ 7 | 241 | 79.8 | 0.0 | 360.0 | 1040.0 |  | 0.0 | 90.0 | 240.0 |  |
| **Fasting plasma glucose level (mg/dL)** |  |  |  |  |  |  |  |  |  |  |
| < 130 | 61 | 20.2 | 400.0 | 1216.0 | 2200.0 | < 0.001 | 65.0 | 180.0 | 385.0 | 0.001 |
| ≥ 130 | 241 | 79.8 | 0.0 | 360.0 | 996.0 |  | 0.0 | 90.0 | 220.0 |  |
| **Postprandial plasma glucose level (mg/dL)** |  |  |  |  |  |  |  |  |  |  |
| < 180 | 81 | 26.8 | 400.0 | 1000.0 | 1940.0 | < 0.001 | 70.0 | 180.0 | 360.0 | < 0.001 |
| ≥ 180 | 221 | 73.2 | 0.0 | 320.0 | 918.0 |  | 0.0 | 75.0 | 210.0 |  |

^*^Calculated based on the number of patients who had health insurance, T2DM: type 2 diabetes mellitus, HbA_1c_: hemoglobin A_1c_

**Supplementary Table S3:** Associations between demographic and disease variables of the patients with the MET class

|  | **MET Class** | | | | | | | |
| --- | --- | --- | --- | --- | --- | --- | --- | --- |
|  | **Low** | | **Moderate** | | **High** | |  |  |
| **Variable** | **n** | **%** | **n** | **%** | **n** | **%** | **Chi-square** | **p-value** |
| **Age (years)** |  |  |  |  |  |  |  |  |
| < 58 | 60 | 19.9 | 54 | 17.9 | 25 | 8.3 | 35.6 | < 0.001 |
| ≥ 58 | 125 | 41.4 | 27 | 8.9 | 11 | 3.6 |  |  |
| **Marital status** |  |  |  |  |  |  |  |  |
| Single | 17 | 5.6 | 9 | 3.0 | 3 | 1.0 | 1.3 | 0.866 |
| Married | 149 | 49.3 | 67 | 22.2 | 30 | 9.9 |  |  |
| Divorced/widowed | 19 | 6.3 | 5 | 1.7 | 3 | 1.0 |  |  |
| **Employment status** |  |  |  |  |  |  |  |  |
| Unemployed | 127 | 42.1 | 26 | 8.6 | 7 | 2.3 | 48.7 | < 0.001 |
| Employed | 58 | 19.2 | 55 | 18.2 | 29 | 9.6 |  |  |
| **Place of residence** |  |  |  |  |  |  |  |  |
| City | 95 | 31.5 | 51 | 16.9 | 15 | 5.0 | 6.6 | 0.155 |
| Village | 68 | 22.5 | 25 | 8.3 | 18 | 6.0 |  |  |
| Refugees camp | 22 | 7.3 | 5 | 1.7 | 3 | 1.0 |  |  |
| **Educational level** |  |  |  |  |  |  |  |  |
| School | 151 | 50.0 | 43 | 14.2 | 22 | 7.3 | 26.1 | < 0.001 |
| Undergraduate | 28 | 9.3 | 34 | 11.3 | 11 | 3.6 |  |  |
| Postgraduate | 6 | 2.0 | 4 | 1.3 | 3 | 1.0 |  |  |
| **Household monthly income** |  |  |  |  |  |  |  |  |
| Low (< 3000 NIS) | 141 | 46.7 | 36 | 11.9 | 14 | 4.6 | 40.5 | < 0.001 |
| Medium (3000-5000 NIS) | 27 | 8.9 | 37 | 12.3 | 14 | 4.6 |  |  |
| High (> 5000 NIS) | 17 | 5.6 | 8 | 2.6 | 8 | 2.6 |  |  |
| **Type of medical insurance*** |  |  |  |  |  |  |  |  |
| Governmental | 174 | 57.6 | 71 | 23.5 | 32 | 10.6 | 1.6 | 0.266 |
| Private | 10 | 3.3 | 9 | 3.0 | 3 | 1.0 |  |  |
| **Participated in an awareness program related to T2DM** |  | 0.0 |  | 0.0 |  | 0.0 |  |  |
| No | 125 | 41.4 | 29 | 9.6 | 15 | 5.0 | 26.5 | < 0.001 |
| Yes | 60 | 19.9 | 52 | 17.2 | 21 | 7.0 |  |  |
| **Received education about the importance of physical activity in T2DM management** |  |  |  |  |  |  |  |  |
| No | 108 | 35.8 | 25 | 8.3 | 13 | 4.3 | 19.5 | < 0.001 |
| Yes | 77 | 25.5 | 56 | 18.5 | 23 | 7.6 |  |  |
| **Physically active before diagnosis** |  |  |  |  |  |  |  |  |
| No | 117 | 38.7 | 32 | 10.6 | 15 | 5.0 | 15.4 | < 0.001 |
| Yes | 68 | 22.5 | 49 | 16.2 | 21 | 7.0 |  |  |
| **Thought that physical activity was crucial for patients with T2DM** |  |  |  |  |  |  |  |  |
| No | 47 | 15.6 | 1 | 0.3 | 0 | 0.0 | 32.3 | < 0.001 |
| Yes | 138 | 45.7 | 80 | 26.5 | 36 | 11.9 |  |  |
| **Have hyperlipidemia** |  |  |  |  |  |  |  |  |
| No | 51 | 16.9 | 44 | 14.6 | 24 | 7.9 | 29.6 | < 0.001 |
| Yes | 134 | 44.4 | 37 | 12.3 | 12 | 4.0 |  |  |
| **Have hypertension** |  |  |  |  |  |  |  |  |
| No | 41 | 13.6 | 49 | 16.2 | 19 | 6.3 | 40.8 | < 0.001 |
| Yes | 144 | 47.7 | 32 | 10.6 | 17 | 5.6 |  |  |
| **Have other health conditions** |  |  |  |  |  |  |  |  |
| No | 94 | 31.1 | 67 | 22.2 | 29 | 9.6 | 30.0 | < 0.001 |
| Yes | 91 | 30.1 | 14 | 4.6 | 7 | 2.3 |  |  |
| **Time since diagnosis (years)** |  |  |  |  |  |  |  |  |
| < 10 | 78 | 25.8 | 58 | 19.2 | 22 | 7.3 | 20.8 | < 0.001 |
| ≥ 10 | 107 | 35.4 | 23 | 7.6 | 14 | 4.6 |  |  |
| **Body mass index class (kg/m^2^)** |  |  |  |  |  |  |  |  |
| Normal weight | 34 | 11.3 | 26 | 8.6 | 19 | 6.3 | 39.1 | < 0.001 |
| Overweight | 53 | 17.5 | 34 | 11.3 | 14 | 4.6 |  |  |
| Obese | 98 | 32.5 | 21 | 7.0 | 3 | 1.0 |  |  |
| **HbA_1c_ (%)** |  |  |  |  |  |  |  |  |
| < 7 | 25 | 8.3 | 19 | 6.3 | 14 | 4.6 | 13.8 | 0.001 |
| ≥ 7 | 160 | 53.0 | 62 | 20.5 | 22 | 7.3 |  |  |
| **Fasting plasma glucose level (mg/dL)** |  |  |  |  |  |  |  |  |
| < 130 | 24 | 7.9 | 21 | 7.0 | 16 | 5.3 | 20.8 | < 0.001 |
| ≥ 130 | 161 | 53.3 | 60 | 19.9 | 20 | 6.6 |  |  |
| **Postprandial plasma glucose level (mg/dL)** |  |  |  |  |  |  |  |  |
| < 180 | 32 | 10.6 | 31 | 10.3 | 18 | 6.0 | 23.8 | < 0.001 |
| ≥ 180 | 153 | 50.7 | 50 | 16.6 | 18 | 6.0 |  |  |

^*^Calculated based on the number of patients who had health insurance, T2DM: type 2 diabetes mellitus, HbA_1c_: hemoglobin A_1c_

**Supplementary Table S4:** Correlation matrix between the continuous variables

|  |  | **Age** | **Time elapsed since diagnosis** | **Body mass index** | **Fasting plasma glucose level** | **Postprandial plasma glucose level** | **HbA_1c_** | **Total physical activity** | **MET** |
| --- | --- | --- | --- | --- | --- | --- | --- | --- | --- |
| **Age** | Spearman's rho | - | 0.56 | 0.18 | 0.23 | 0.26 | 0.24 | -0.38 | -0.35 |
|  | p-value |  | < 0.001 | 0.002 | < 0.001 | < 0.001 | < 0.001 | < 0.001 | < 0.001 |
| **Time elapsed since diagnosis** | Spearman's rho | 0.56 | - | 0.28 | 0.29 | 0.35 | 0.29 | -0.34 | -0.27 |
|  | p-value | < 0.001 |  | < 0.001 | < 0.001 | < 0.001 | < 0.001 | < 0.001 | < 0.001 |
| **Body mass index** | Spearman's rho | 0.18 | 0.28 | - | 0.37 | 0.39 | 0.32 | -0.38 | -0.28 |
|  | p-value | 0.002 | < 0.001 |  | < 0.001 | < 0.001 | < 0.001 | < 0.001 | < 0.001 |
| **Fasting plasma glucose level** | Spearman's rho | 0.23 | 0.29 | 0.37 | - | 0.93 | 0.78 | -0.40 | -0.32 |
|  | p-value | < 0.001 | < 0.001 | < 0.001 |  | < 0.001 | < 0.001 | < 0.001 | < 0.001 |
| **Postprandial plasma glucose level** | Spearman's rho | 0.26 | 0.35 | 0.39 | 0.93 | - | 0.79 | -0.42 | -0.33 |
|  | p-value | < 0.001 | < 0.001 | < 0.001 | < 0.001 |  | < 0.001 | < 0.001 | < 0.001 |
| **HbA_1c_** | Spearman's rho | 0.24 | 0.29 | 0.32 | 0.78 | 0.79 | - | -0.36 | -0.28 |
|  | p-value | < 0.001 | < 0.001 | < 0.001 | < 0.001 | < 0.001 |  | < 0.001 | < 0.001 |
| **Total physical activity** | Spearman's rho | -0.38 | -0.34 | -0.38 | -0.40 | -0.42 | -0.36 | - | 0.92 |
|  | p-value | < 0.001 | < 0.001 | < 0.001 | < 0.001 | < 0.001 | < 0.001 |  | < 0.001 |
| **MET** | Spearman's rho | -0.35 | -0.27 | -0.28 | -0.32 | -0.33 | -0.28 | 0.92 | - |
|  | p-value | < 0.001 | < 0.001 | < 0.001 | < 0.001 | < 0.001 | < 0.001 | < 0.001 |  |
